# Supplementary material for: HIVprotI: an integrated web based platform for prediction and design of HIV proteins inhibitors
Source: J Cheminform. 2018 Mar 9;10:12. doi: 10.1186/s13321-018-0266-y (PMC5845081; doi:10.1186/s13321-018-0266-y)
Supplement: Supplementary file 2 — Additional file 2. Source code of HIVProtI web server. [file 13321_2018_266_MOESM2_ESM.zip › HIVprotI_Source-code/hivprotI/weka-3-6-6/doc/weka/gui/treevisualizer/TreeDisplayListener.html]

TreeDisplayListener


---


|  |  |  |
| --- | --- | --- |
| **Overview** \| **Package** \| **Class** \| **Tree** \| **Deprecated** \| **Index** \| **Help** \| **Weka's home** | |  |
| **PREV CLASS**   **NEXT CLASS** | **FRAMES**    **NO FRAMES**     **All Classes** |  |
| SUMMARY: NESTED \| FIELD \| CONSTR \| METHOD | DETAIL: FIELD \| CONSTR \| METHOD |  |


---


## weka.gui.treevisualizer Interface TreeDisplayListener

**All Known Implementing Classes:**: UserClassifier

---

``` public interface TreeDisplayListener ```

Interface implemented by classes that wish to recieve user selection events
from a tree displayer.

**Version:**
:   $Revision: 1.5 $

**Author:**
:   Malcolm Ware (mfw4@cs.waikato.ac.nz)

---

| **Method Summary** | |
| --- | --- |
| `void` | `userCommand(TreeDisplayEvent e)`             Gets called when the user selects something, in the tree display. |

**Method Detail**

### userCommand

```
void userCommand(TreeDisplayEvent e)
```

:   Gets called when the user selects something, in the tree display.

    :   **Parameters:**: `e` - Contains what the user selected with what it was selected for.


---


|  |  |  |
| --- | --- | --- |
| **Overview** \| **Package** \| **Class** \| **Tree** \| **Deprecated** \| **Index** \| **Help** \| **Weka's home** | |  |
| **PREV CLASS**   **NEXT CLASS** | **FRAMES**    **NO FRAMES**     **All Classes** |  |
| SUMMARY: NESTED \| FIELD \| CONSTR \| METHOD | DETAIL: FIELD \| CONSTR \| METHOD |  |


---
